# Supplementary material for: PPyNT/NR/NBR Composites with Excellent Microwave Absorbing Performance in X-Band
Source: Polymers (Basel). 2023 Apr 13;15(8):1866. doi: 10.3390/polym15081866 (PMC10142120; doi:10.3390/polym15081866)
Supplement: Supplementary file 1 [file polymers-15-01866-s001.zip › polymers-2332740-supplementary.pdf]

Supplementary Materials: PPyNT/NR/NBR Composites with Excellent Microwave Absorbing Performance in X-Band

Huiru Yang [1], Aiping Wang [2], Xincong Feng [1], Hailing Dong [1], Tao Zhuang [1], Jing Sui [1,*] and Shugao Zhao [1] and Chong Sun [1,*]

[1] Key Laboratory of Rubber-Plastics, Ministry of Education/Shandong Provincial Key Laboratory of Rubber-Plastics, Qingdao University of Science & Technology, Qingdao 266042, China; yhr1150286872@163.com (H.Y.)

[2] School of Materials Science and Engineering, Ocean University of China, Qingdao 266100, China; chunxiaz@126.com

* Correspondence: jingsui1007@qust.edu.cn (J.S.); sunchongmc@qust.edu.cn (C.S.)

Equations:

The reflection loss (*RL*) values are calculated according to the following equations:

$$RL=20\lg\left|\frac{Z_{in}-Z_0}{Z_{in}+Z_0}\right| \quad (S1)$$

$$Z_{in}=Z_0\sqrt{\frac{\mu_r}{\varepsilon_r}}\tanh\left(\frac{2\pi fdj}{c}\times\sqrt{\mu_r\varepsilon_r}\right) \quad (S2)$$

in which Z_{in} and Z_0 stand for the normalized input impedance of the material and impedance of free space respectively, while μ_r, ε_r, f, c, d are the complex relative permeability, the complex relative permittivity, the microwave frequency, the velocity of electromagnetic wave in vacuum and the thickness of the material respectively.

The attenuation constant (α) is calculated by using the following equation:

$$\alpha=\frac{\sqrt{2}\pi f}{c}\times\sqrt{(\mu''\varepsilon''-\mu'\varepsilon')+\sqrt{(\mu''\varepsilon''-\mu'\varepsilon')^2+(\mu''\varepsilon''+\mu'\varepsilon')^2}} \quad (S3)$$

The impedance matching $|Z_{in}/Z_0|$ is calculated as following

$$Z_{in}/Z_0=\sqrt{\frac{\mu_r}{\varepsilon_r}}\tanh\left(\frac{2\pi fdj}{c}\times\sqrt{\mu_r\varepsilon_r}\right) \quad (S4)$$

Debye relaxation (Cole-Cole curves) are described using the following equation:

$$\left(\varepsilon'-\frac{\varepsilon_s+\varepsilon_\infty}{2}\right)^2+\left(\varepsilon''\right)^2=\left(\frac{\varepsilon_s-\varepsilon_\infty}{2}\right)^2 \quad (S5)$$

in which ε_s and ε_∞ are the static dielectric constant and the dielectric constant at infinite frequency, respectively.

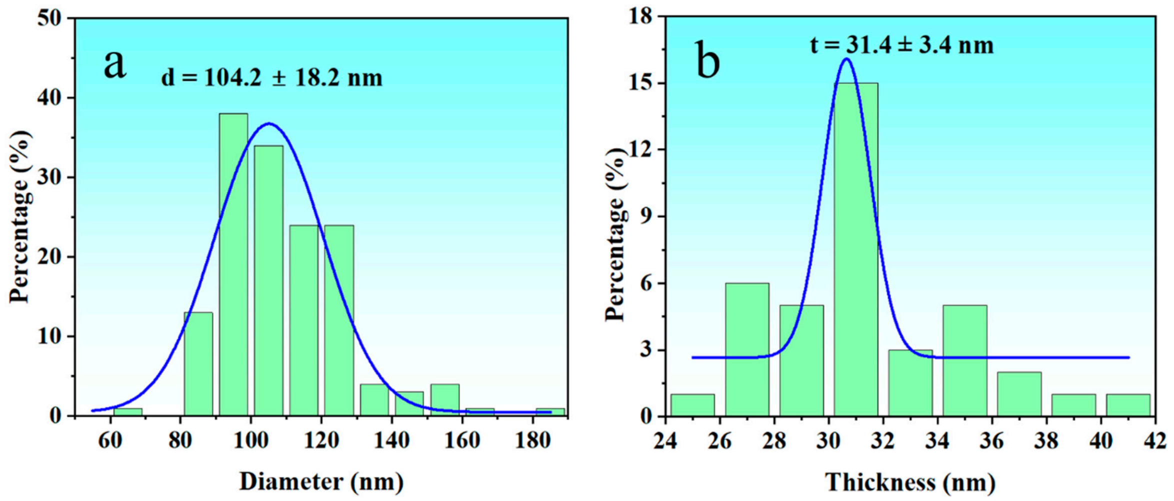

Figure S1. (a) Diameter and **(b)** Wall thickness distribution of PPyNTs (n= 150)

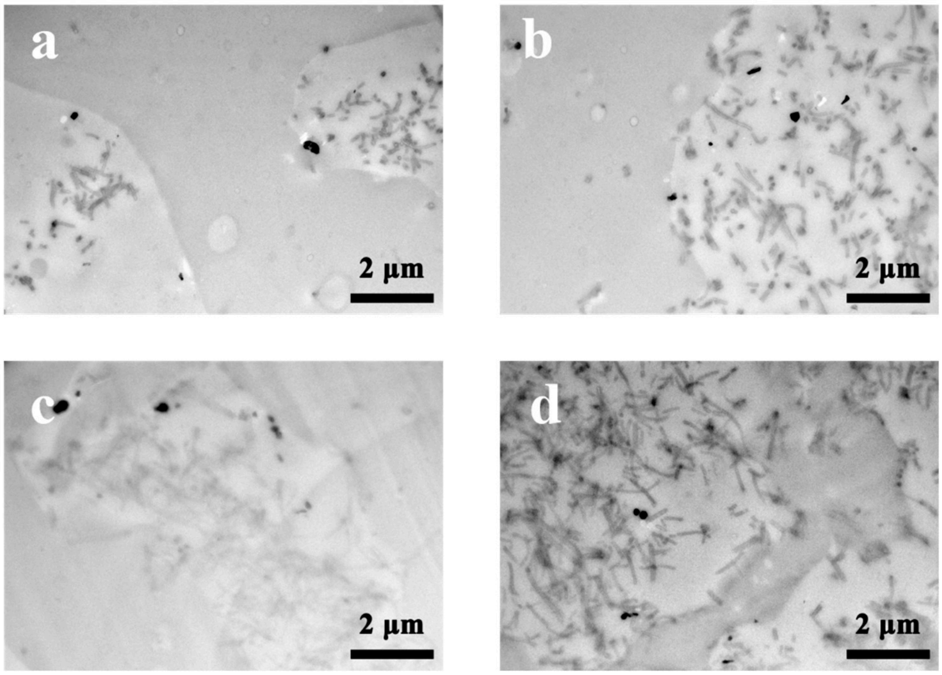

Figure S2. TEM images of PPyNT/NR/NBR (50/50) with different PPyNT contents **(a)** 3 phr; **(b)** 6 phr; **(c)** 9 phr; **(d)** 12 phr.

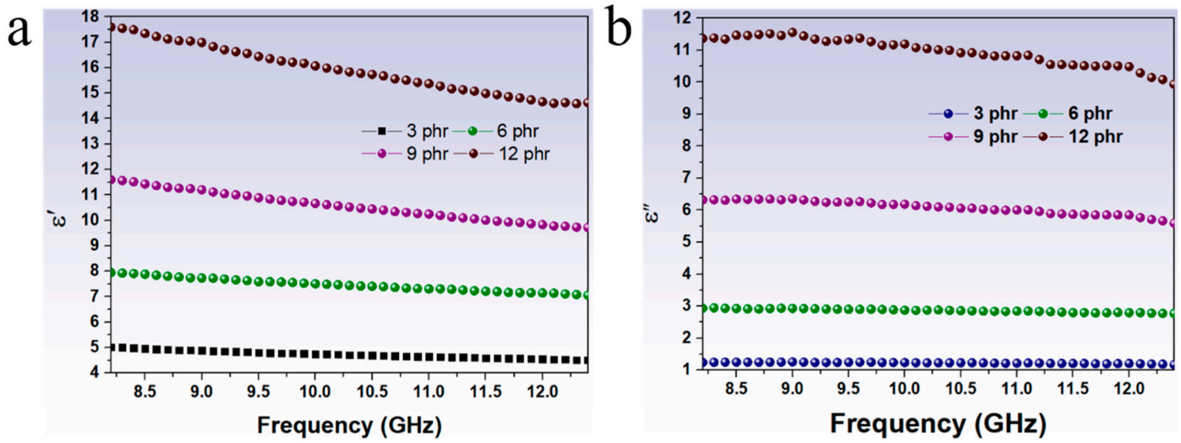

Figure S3. The real ε′ **(a)** and imaginary ε" **(b)** parts of the complex permittivity of PPyNT/NR/NBR (50/50) with different PPyNT contents.

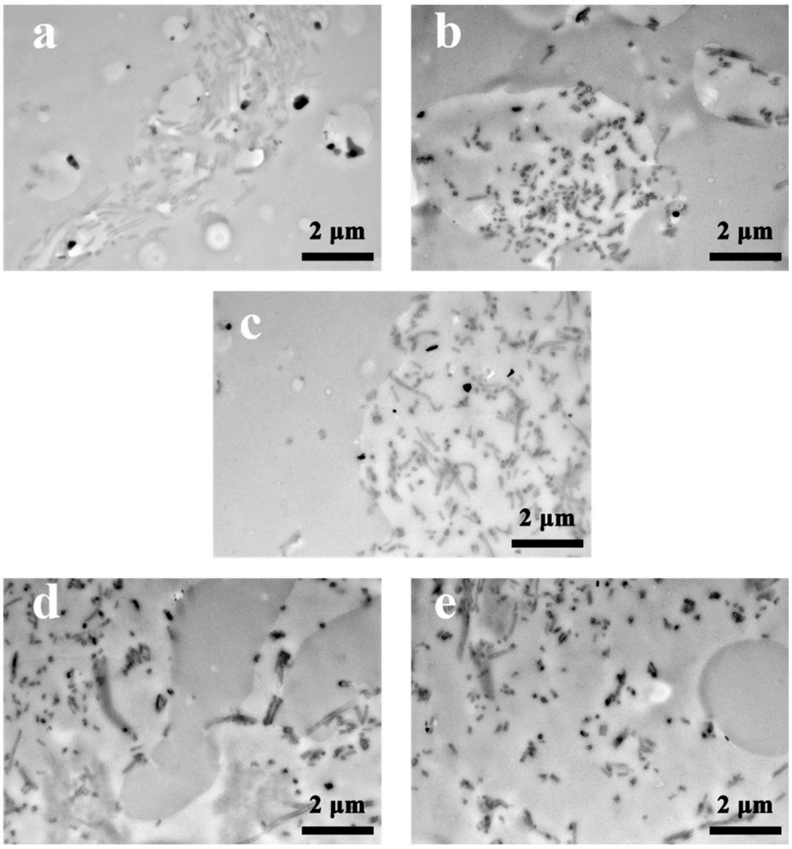

Figure S4. TEM images of 6 phr PPyNT filled NR/NBR composites with different ratios of NR/NBR: **(a)** 10/90; **(b)** 30/70; **(c)** 50/50; **(d)** 70/30; **(e)** 90/10.

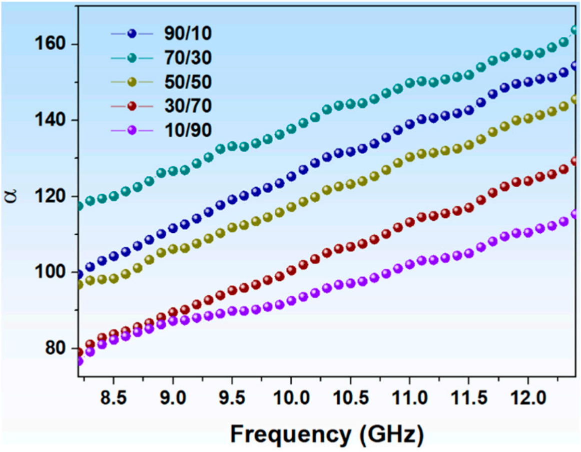

Figure S5. Attenuation constant of 6 phr PPyNT filled NR/NBR composites with different ratios of NR/NBR.

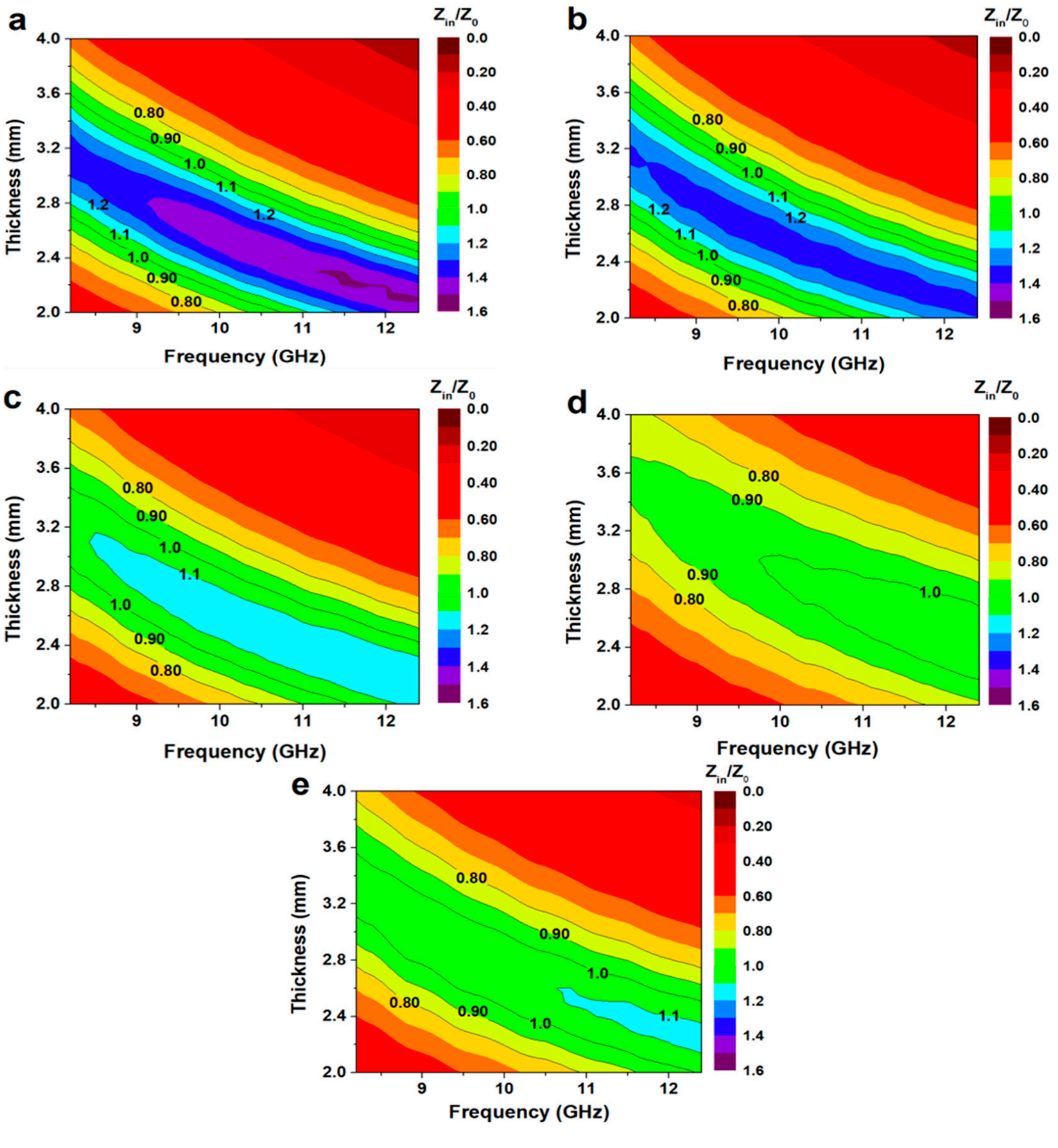

Figure S6. 2D contour maps of $|Z_{in}/Z_0|$ values of 6 phr PPyNT filled NR/NBR composites with different ratios of NR/NBR: **(a)**10/90; **(b)** 30/70; **(c)** 50/50; **(d)** 70/30; **(f)** 90/10.
